# Forearm vasodilator responses to environmental stress and reactive hyperaemia are impaired in young South Asian men

**DOI:** 10.1007/s00421-018-3829-8

**Published:** 2018-03-03

**Authors:** Natalie G. Ormshaw, Rehan T. Junejo, Janice M. Marshall

**Affiliations:** Institute of Cardiovascular Science, College of Medical and Dental Sciences, The Medical School, University of Birmingham, Birmingham, B15 2TT UK

**Keywords:** Reactive hyperaemia, Forearm dilatation, Mental stress, South Asian

## Abstract

**Purpose:**

Prevalence of cardiovascular disease (CVD) is greater in South Asians (SAs) than White Europeans (WEs). Endothelial dysfunction and blunted forearm vasodilatation to environmental stressors have been implicated in CVD. We investigated whether these features are present in young SA men.

**Methods:**

In 15 SA and 16 WE men (19–23 years), we compared changes in forearm blood flow, arterial blood pressure (ABP), forearm vascular conductance (FVC), heart rate, and electrodermal resistance (EDR; sweating) following release of arterial occlusion (reactive hyperaemia endothelium-dependent) and 5 single sounds at 5–10 min intervals (stressors).

**Results:**

All were normotensive. Peak reactive hyperaemia was smaller in SAs than WEs (FVC increase: 0.36 ± 0.038 vs 0.44 ± 0.038 units; *P* < 0.05). Furthermore, in WEs, mean FVC increased at 5, 15, and 20 s of each sound (vasodilatation), but increased at 5 s only in SAs, decreasing by 20 s (vasoconstriction). This reflected a smaller proportion of SAs showing forearm vasodilatation at 15 s (5/15 SAs vs 11/16 WEs: *P* < 0.01), the remainder showing vasoconstriction. Concomitantly, WEs showed greater bradycardia and EDR changes. Intra-class correlation analyses showed that all responses were highly reproducible over five sounds in both WEs and SAs. Moreover, sound-evoked changes in ABP and FVC were negatively correlated in each ethnicity (*P* < 0.01). However, WEs showed preponderance of forearm vasodilatation and depressor responses; SAs showed preponderance of vasoconstriction and pressor responses.

**Conclusions:**

Endothelium-dependent vasodilatation is blunted in young SA men. This could explain their impaired forearm vasodilatation and greater pressor responses to repeated environmental stressors, so predisposing SAs to hypertension and CVD.

## Introduction

Population studies in the UK, Canada and USA indicate that South Asians (SAs) with ethnic roots in India, Pakistan, Bangladesh, Sri Lanka, or Nepal, have a 2–3-fold greater risk of death from all cardiovascular disease (CVD) than those of White European origin (WE; Gupta et al. 2006; Rana et al. 2014; Wild et al. 2007). Moreover, SAs present at younger age with coronary artery disease and heart failure and have higher prevalence of hypertension, the common forerunner of CVD (Agyemand and Bhopal 2006; Gupta et al. 2006). The prevalence of type 2 diabetes is also higher in SAs than WE (Gupta et al. 2006). However, SA men and women (20–60 years) in the USA, who were non-diabetic and normotensive had higher plasma insulin and showed blunted brachial artery vasodilatation to insulin relative to age-matched WEs (Raji et al. 2004). Furthermore, non-diabetic, normotensive SA men (20–40 years) resident in the UK showed higher plasma insulin, smaller flow-mediated dilatation (FMD) and smaller tonic dilator influence of nitric oxide (NO) than WEs (Murphy et al. 2007). These findings suggest that endothelial dilator function deteriorates at an earlier age in SA than WE men and may precede development of hypertension and diabetes.

It is widely accepted that psychosocial stress is an important “modifiable” risk factor for CVD as identified in the INTERHEART study of acute myocardial infarction in 52 countries (Yusuf et al. 2004). Consistent with this, meta-analysis of longitudinal studies of cardiovascular responses to laboratory mental stress and future cardiovascular status demonstrated that greater pressor reactivity to mental stress in young individuals predicted future development of hypertension (Chida and Steptoe 2010). Whether enhanced pressor responses to mental stress and hypertension are causally linked is controversial (Fonkoue and Carter 2015; Lambert and Schlaich 2004; Oparil et al. 2003). However, in normotensives, a single episode of mental stress attenuated endothelium-dependent dilatation in forearm for at least 4 h (Ghiadoni et al. 2002). Furthermore, the pattern of vascular response evoked by acute mental stress includes muscle vasodilatation (Brod et al. 1959; Halliwill et al. 1997; Hilton 1982) and this vasodilatation is labile in a way that seems likely to facilitate development of hypertension in some individuals. Thus, muscle vasodilator responses evoked by stress tests habituated on repetition and reversed to vasoconstriction in some individuals (Edwards et al. 1998, 1999; Zbrozyna and Westwood 1988). Furthermore, a decrease in muscle sympathetic nerve activity (MSNA) occurred in ~ 50% of individuals and habituated on repetition of the stress stimulus. Recurrent increases in MSNA occurred in the remainder and they showed larger and more persistent increases in arterial blood pressure (Donadio et al. 2002, 2012). Similarly, young borderline hypertensives with hypertensive parents showed greater pressor responses to mental stress and smaller decreases in MSNA than young normotensive men with normotensive parents (Matsukawa et al. 1991; Fonkoue et al. 2016). Moreover, mental stress evoked smaller forearm vasodilator responses in young borderline hypertensives than normotensives, while muscle vasodilator responses habituated in young normotensives with hypertensive parents and in borderline hypertensives (Schneider et al. 2003; Schwartz et al. 2011; Zbrozyna and Krebbel 1985).

In view of these findings, we hypothesised that the forearm vasodilator response to a recognised sound stress stimulus (see Edwards et al. 1998, 1999) would be smaller and habituate more readily on repetition in young SAs than young WEs, while other components would be larger. We also hypothesised that endothelium-dependent, reactive hyperaemia in forearm would be smaller in SAs than WEs. A brief account of our results has been published in abstract form (Ormshaw et al. 2016).

## Methods

The study was performed on 31 male subjects aged 20.80 ± 0.16 years (mean ± SEM, range 19–23) of either South Asian (SA, *n* = 15), or White European (WE, *n* = 16) ethnicity. Ethnicity was self-reported (Office for National Statistics 2018); all were UK residents with both parents of the same ethnicity. One subject in each ethnic group had type 1 diabetes; otherwise, they had no known medical condition. They were all non-smokers, except one in each group who had quit smoking for at least 12 months. All experiments were performed in accordance with the Helsinki Declaration and approved by the University of Birmingham Ethics Committee. Subjects were recruited from students of the University of Birmingham, UK and gave written, informed consent.

Each subject attended a preliminary session during which he was familiarized with the recording equipment, completed Cohen’s Perceived Stress Scale questionnaire (PSS; Cohen et al. 1983) and a questionnaire dealing with personal characteristics. We used a semi-quantitative scoring system based on the UK Diabetes and Diet Questionnaire (UKDDQ) for fresh fruit and vegetable consumption, as an index of anti-oxidant vitamin C intake. Alcohol consumption was scored as units/week. Level of elective physical activity was assessed according to the International Physical Activity Questionnaire short version, allowing subjects to indicate time spent in moderate and vigorous exercise per week and scored as MET min/week (The IPAQ Group 2015). Time spent in dynamic exercise (e.g., running, jogging) vs resistance exercise (e.g., press-ups, dumbbell lift) was calculated as percentage of total time. In addition, measurements of resting arterial blood pressure (ABP) were taken with a sphygmomanometer.

### Experimental protocol

Subjects were asked to refrain from consuming alcohol, a heavy meal and undertaking vigorous exercise for at least 24 h prior to the experiment. Experiments were performed in a quiet room with the subject sitting on a couch, with the backrest inclined at 45° and facing away from the equipment to avoid distractions. Both arms were supported at heart level. ABP was continuously recorded from the right middle finger using a Finapres (Ohmeda 2300, BOC Health Care). Forearm blood flow (FBF) was recorded by venous occlusion plethysmography (strain gauge and gadolinium-filled silastic tubing; EC6, Hokanson Inc.) following inflation of a cuff around the upper arm cuff to 50 mmHg using a rapid inflation system (AG101 Cuff Inflator Air Source; E20 Rapid Cuff Inflator Hokanson Inc.). Values of FBF were computed off-line as the slope over the first two heartbeats following venous occlusion. A second cuff wrapped around the wrist was inflated to 200 mmHg immediately prior to each recording of FBF to exclude blood flow to the hand. Forearm vascular conductance (FVC) was calculated as FBF/mean ABP. Electrodermal response (EDR) was recorded as an index of sweating, via an Amplifier (GSR Amp FE116; AD Instruments), by electrodes (MLT116; AD Instruments) attached to the first and third finger of the right hand, as maximum change from the baseline. Mean ABP and heart rate (HR) generated from the Finapres recordings, FBF and EDR were recorded to a computer via MacLab software (PowerLab/85P, AD instruments).

### Reactive hyperaemia

After a stabilization period of 15 min, four baseline measurements of FBF were taken at 30 s intervals. The upper arm cuff was then inflated to 200 mmHg for 2 min. At 2 min, the upper arm cuff was rapidly deflated: FBF was measured immediately and at 15, 30, 60, 90, and 120 s post-occlusion.

### Repeated sounds

The subject closed his eyes for this part of the protocol to prevent anticipatory stress that might have been triggered by the subject seeing the experimenter initiate the sound stimulus. Five consecutive sounds (S1–S5: 100 dB, 1.5 kHz; Edwards et al. 1998) each of 30 s duration were delivered through headphones by an audiometer (Maico MA40, Graystad, Medical), at randomized intervals of 5–10 min. FBF was recorded at 5, 15, and 20 s into each sound: 5 s was the earliest timepoint at which we could measure and gave us an index of the very early part of the response when ABP is rising; 15 s is the timepoint we have used before when ABP and skin vasoconstriction have reached their peak (Edwards et al. 1998); a measurement at 20 s was included to indicate whether the forearm vascular response stabilised. The subject rated each sound on a scale of 0–10, 10 being the most stressful.

### Statistical analyses

Results are expressed as mean ± SEM. Baseline comparisons between ethnicities were made with Student’s un-paired *t* test. Proportions of subjects with hypertensive vs normotensive parents, those born in UK vs non-UK and time spent in dynamic vs resistance exercise were compared by Fisher’s exact test. For reactive hyperaemia, within and between group analyses were performed by two-way repeated measures ANOVA. Responses evoked by sound were compared by mixed model ANOVA with repeated measures. Post-hoc comparisons were made with Tukey’s HSD for multivariate comparisons. Individuals were divided into “vasodilators” and “vasoconstrictors” according to the direction of change in FVC at 15 s into S1 when the evoked change in ABP and cutaneous vasoconstriction reach their maximum (Edwards et al. 1998). The proportions of vasodilators and vasoconstrictors amongst WEs and SAs were compared by Fisher’s exact test. The consistency of changes evoked in each variable at 15 s into the five sounds was tested by intra-class correlation (ICC) analysis; the coefficient of consistency [Cronbach’s alpha (*α*) value] is reported with two-tailed *P* values. Relationships between variables within individuals of each ethnicity were investigated by linear regression analysis using Pearson’s correlation coefficient (*r*) and coefficient of determination (*r*^2^). *P* < 0.05 was taken to indicate statistical significance.

## Results

### Baseline characteristics

There were no significant differences between the ethnic groups for anthropometric data and lifestyle variables except caffeine intake, which was greater in WEs (Table 1); it should be noted that all subjects refrained from caffeine intake for at least 24 h before the experiment. There were also no differences between ethnic groups for resting ABP, or cardiovascular baselines recorded at the beginning of the experimental protocol (Table 2).

**Table 1:** Characteristics of South Asians and White Europeans

	South Asians (*n* = 15)	White Europeans (*n* = 16)	*P* value
Age (years)	20.6 ± 0.214	21.1 ± 0.232	0.155
BMI (kg/m^2^)	22.5 ± 0.754	21.9 ± 0.578	0.529
Exercise (MET min/week)	2702.2 ± 435.526	2094 ± 278.779	0.257
Dynamic: resistive exercise (%)	74:26	79:21	0.094
Vit C-containing portions/day	5.87 ± 0.274	6.69 ± 0.463	0.117
Caffeine intake (cups/day)	0.533 ± 0.236	1.81 ± 0.410	0.003
Parental hypertension (Y/N)	4/11	3/13	0.291
UK birth (Y/N)	12/3	15/1	0.239
SBP (mmHg)	124 ± 2.55	122 ± 1.78	0.578
DBP (mmHg)	74.6 ± 1.76	73.0 ± 1.73	0.536
MABP (mmHg)	90.8 ± 1.93	89.8 ± 1.55	0.399
HR (beats/min)	78.2 ± 2.88	70.7 ± 3.19	0.0843

All values are mean ± SEM unless otherwise stated. *P* value: WEs vs SAs. For further description of scoring systems see text

*BMI* body mass index, *HR* heart rate, *SBP* systolic blood pressure, *DBP* diastolic blood pressure, *MABP* mean arterial blood pressure, *exercise* elective physical activity scored as MET min/week spent in moderate exercise and vigorous exercise, *Vit C-containing portions* high vitamin C-containing fruit and vegetable portions

**Table 2:** Baseline values recorded in South Asians and White Europeans

	South Asians	White Europeans	*P* value
MABP (mmHg)	82.1 ± 3.4	88.8 ± 3.5	0.184
HR (beats/min)	66.5 ± 2.3	67.8 ± 2.4	0.703
FBF (ml 100/ml/min)	6.90 ± 0.724	6.16 ± 0.661	0.461
FVC (CU)	0.088 ± 0.012	0.069 ± 0.007	0.166

*P* value: WEs vs SAs

*MABP* mean arterial blood pressure, *HR* heart rate, *FBF* forearm blood flow, *FVC* forearm vascular conductance, *CU* conductance units

### Reactive hyperaemia

**Fig. 1:**
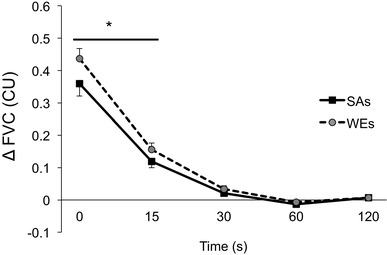
Reactive hyperaemia evoked in forearm of White Europeans (WEs) and South Asians (SAs). Solid black symbols: SAs. Grey filled symbols: WEs. *P < 0.05 WEs vs SAs

In SAs and WEs, FVC was increased at time 0 and returned to baseline by 2 min following release of occlusion (*P* < 0.01 in each case). The increase in FVC was significantly greater in the WEs than SAs at 0 and 15 s (*P* = 0.012; 0.014, respectively, Fig. 1).

### Responses evoked by sound

**Fig. 2:**
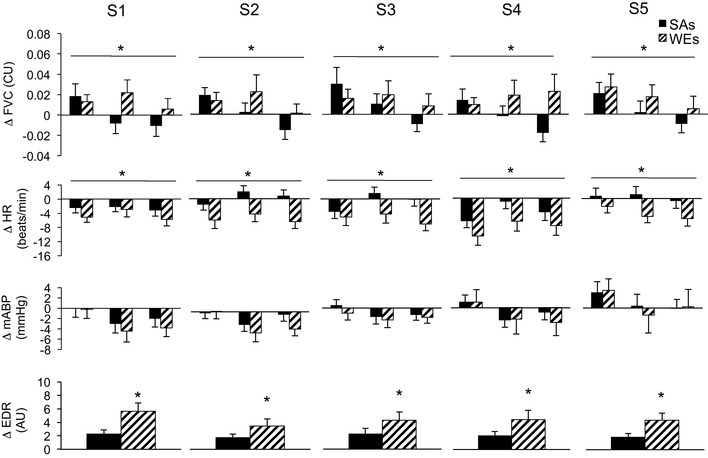
Cardiovascular and sweating responses evoked in response to sounds 1–5 in South Asians (SAs) and White Europeans (WEs). Cardiovascular variables were measured at 5, 15, and 20 s into each sound, whereas sweating (EDR) was averaged over 30 s (for further details, see text). Solid black columns: SAs. Cross-hatched columns: WEs. *WE vs SA, *P* < 0.05

Considering group mean values (Fig. 2), the variability was high in both groups, but the pattern of response evoked by each sound stimulus (S1–S5) differed significantly between SAs and WEs (*P* < 0.05 for all variables except ABP). Thus, WEs showed a mean increase in FVC at 5, 15, and 20 s into each sound, whereas SAs showed a mean increase in FVC at 5 s, with a decrease in FVC by 20 s (*P* = 0.007 for WE vs SA across S1–S5; *P* < 0.05 for each sound). There was no significant difference between WE and SA for change in ABP across S1–S5 (*P* = 0.74), but WEs showed a greater decrease in HR across S1–S5 (*P* = 0.01; *P* < 0.05 for each sound). EDR was consistently greater in WEs than SAs (*P* = 0.01 across S1–S5; *P* < 0.05 for each sound).

Despite the group variability, changes evoked by S1–S5 within individuals were reproducible. The majority of WEs and SAs showed an increase in FVC at 5 s (11/16 and 11/15, respectively). However, by 15 s of S1, a greater proportion of WEs than SEs showed an increase in FVC (dilatation; 11/16 vs 5/15, respectively, Fisher’s exact test: *P* = 0.006). Correspondingly, a greater proportion of SAs showed a decrease in FVC (constriction; 10/15 vs 5/16). ICC analysis revealed that the reproducibility of responses at 15 s was high in individuals amongst both WEs and SAs: Cronbach’s *α* for WEs and SAs, respectively, were 0.94 and 0.80 for FVC, 0.94 and 0.86 for ABP, 0.87and 0.88 for HR, and 0.98 and 0.92 for EDR (*P* < 0.001 in each case). In other words, there was no obvious habituation of forearm vascular response with repetition of sounds in either WEs or SAs; within group comparisons for effect of sound repetition on change in FVC; *P* = 0.975 and *P* = 0.938, respectively. In fact, only two WEs who showed an increase in FVC at S1, switched to a substantial decrease in FVC by S4 and S5, consistent with habituation of forearm vasodilatation (Edwards et al. 1998, 1999; Zbrozyna and Westwood 1988); no vasodilator SAs showed such behaviour.

**Fig. 3:**
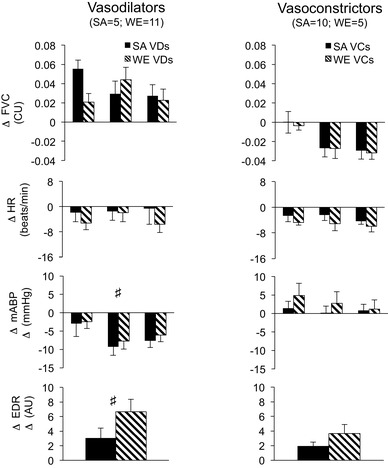
Cardiovascular and sweating responses evoked in response to sound 1 in vasodilator and vasoconstrictor SAs and WEs (left, right, respectively). Solid black columns: SAs. Cross-hatched columns: WEs. Abbreviations and timing of measurements as in Fig. 2. ^#^*P* < 0.05 for changes evoked by S1 in vasodilators vs changes evoked by S1 in vasoconstrictors

When WEs and SAs were categorized as “vasodilators” or “vasoconstrictors” by the direction of change in FVC at 15 s, baselines were not significantly different between the subgroups apart from HR, which was lower in vasoconstrictors (Table 3). However, the pattern of response evoked by S1–S5 was different between vasodilators and vasoconstrictors (see Fig. 3, for S1); data for S2–S5 are not shown, but consistent with the ICC values, and these responses were highly comparable to those of S1. Changes in FVC between the vasodilator and vasoconstrictor subgroups were not compared, because we chose to separate individuals into subgroups on this basis. However, the changes in ABP and EDR differed between vasodilators and constrictors when WEs and SAs were considered together (*P* < 0.001 and *P* = 0.045, respectively, across S1–S5). Mean ABP fell by ~ 5–10 mmHg at 15 s in vasodilators (Fig. 3), but showed a trend to increase by up to 5 mmHg in vasoconstrictors (Fig. 3). EDR responses were greater in vasodilators; any apparent trend for EDR responses to be greater in WEs than SAs was not statistically significantly (*P* = 0.197, 0.153 for vasodilators, vasoconstrictors, respectively).

**Table 3:** Baseline values in South Asian and White European constrictor and dilator subgroups

	South Asians		White Europeans		*P* value
	Dilators (*n* = 5)	Constrictors (*n* = 10)	Dilators (*n* = 11)	Constrictors (*n* = 5)	
MABP (mmHg)	81.0 ± 4.61	82.7 ± 4.75	88.4 ± 4.29	83.6 ± 3.37	0.330
HR (beats/min)	69.6 ± 4.95	65.0 ± 2.57	71.3 ± 2.68	60.2 ± 2.79	0.023
FBF (ml 100/ml/min)	6.08 ± 0.951	7.31 ± 0.982	5.90 ± 0.837	6.74 ± 1.13	0.238
FVC (CU)	0.076 ± 0.014	0.094 ± 0.017	0.063 ± 0.007	0.082 ± 0.015	0.089

*P* value: dilators vs constrictors

*MABP* mean arterial blood pressure, *HR* heart rate, *FBF* forearm blood flow, *FVC* forearm vascular conductance, *CU* conductance units

**Fig. 4:**
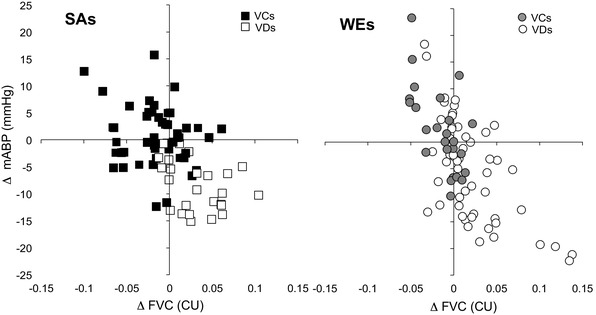
Relationship between evoked change in FVC and ABP in response to each of sounds S1–S5 in individual SAs (left) and WEs (right). In each ethnicity, subjects were categorized as vasoconstrictors (VCs) or vasodilators (VDs) by direction of change in FVC at 15 s into S1

Across S1–S5, the preponderance of repeated forearm vasodilator responses and falls in ABP amongst WEs, but repeated forearm vasoconstrictor responses and rises in ABP amongst SAs was also evident when change in ABP was plotted against change in FVC for each sound in each individual (Fig. 4). In both WEs and SAs, changes in ABP at 15 s were negatively correlated with changes in FVC (*r* = 0.70, *r*^2^ = 0.49; *P* < 0.001 and *r* = 0.43, *r*^2^ = 0.18; *P* = 0.001, respectively).

### Sound stress rating and perceived stress score

Sound stress ratings were greater in WEs than SAs across S1–S5 (*P* = 0.039; mean sound stress rating across S1–S5: 6.19 ± 0.17 vs 5.76 ± 0.19, respectively). The PSS showed a trend to be greater in WEs than SAs (12.5 ± 1.74 vs 8.73 ± 1.17, respectively; *P* = 0.101). There was no correlation between SS score and change in FVC at 15 s in WEs or SAs (WEs: *r* = 0.13, *r*^2^ = 0.02; *P* = 0.26; SAs: *r* = 0.19, *r*^2^ = 0.03; *P* = 0.13).

## Discussion

The new findings of the present study are first that young SA men showed blunted reactive hyperaemia in forearm relative to young WE men. Second, repeated sound stimuli evoked repeatable, maintained forearm vasodilator response in the majority of WEs, whereas most SAs consistently showed transient forearm vasodilatation, which waned to vasoconstriction.

These disparities are noteworthy, because both the SAs and WEs were from a young age range (19–23 years) with no obvious signs of hypertension or other CVD. They had BMI values below those considered a risk for type 2 diabetes and CVD (23 and 25 kg/m^2^, respectively; NICE Guidance 2014). They were also recreationally active, with alcohol consumption well below the recommended limit. The great majority were born in the UK and similar small proportions had hypertensive parents (4/15 SAs; 3/16 WEs). Thus, the only clear difference between these young SA and WEs was their ethnicity.

### Reactive hyperaemia

The blunted reactive hyperaemia in young SA men relative to WE men is fully consistent with our hypothesis. Reactive hyperaemia is attributed to myogenic dilatation, augmented, and maintained by endothelium-dependent vasodilators generated by hypoxia and shear stress (Carlsson et al. 1987; Engelke et al. 1996). It has been reported that NO contributes to peak reactive hyperaemia in normal subjects, but its contribution is attenuated in patients at high risk of CVD (Dakak et al. 1998). Other deduced prostaglandins (PGs), adenosine, and vascular hyperpolarization contribute to the peak, while NO helps maintain reactive hyperaemia (Carlsson et al. 1987; Engelke et al. 1996; Crecelius et al. 2013; Tagawa et al. 1994). Collectively, these findings are consistent with adenosine, PGs and NO acting interdependently (Carlsson et al. 1987; Ray et al. 2002). The tonic vasodilator influence of NO in forearm was attenuated in SAs aged 20–40 years relative to WEs (Murphy et al. 2007). Thus, it seems likely blunted reactive hyperaemia in young SA men reflects blunted contributions of NO, adenosine and/or PGs. Clearly, these proposals should be investigated.

Interestingly, in a longitudinal study of fit, healthy men in the USA, peak reactive hyperaemia in forearm was shown to be a better prognostic indicator of future CVD than FMD of the brachial artery (Anderson et al. 2011). FMD was not depressed in SAs aged 20–40 years (Murphy et al. 2007). Thus, it is reasonable to suggest that the blunted reactive hyperaemia we describe in young SA men may be an early predictor of increased risk of CVD.

### Responses evoked by repeated sound

Vasodilatation in limb muscle is a characteristic component of the behavioural and autonomic pattern of the alerting or defence response to environmental and mental stressors (Brod et al. 1959; Hilton 1982). Accordingly, the group mean data of the present study indicate that young normotensive WE men who were naïve to the sound stimulus, generally showed well-maintained forearm vasodilator responses to repeated sounds with no habituation within session. This compares favorably with our findings on normotensive WE subjects of wider age range (21–70, mean 39 years) who were similarly naïve, but mainly female: the majority showed forearm vasodilatation at 15 s which habituated over 3 sessions, but not within the first session (Edwards et al. 1998).

On the other hand, judging from group mean data, young SA men generally showed only transient forearm vasodilatation that waned to vasoconstriction with each sound. This agrees with our hypothesis that young SA men would show blunted forearm vasodilatation relative to WEs. It was reported that normotensive black African Americans aged ~ 42 years, showed blunted forearm vasodilatation to mental stress relative to White Americans (Cardillo et al. 1998a). We believe that ours is the first evidence that forearm vasodilatation evoked by environmental stress is blunted in young normotensive SA men relative to WE men.

In our previous study, WEs showed a rise in ABP and tachycardia with each sound (Edwards et al. 1998) as is typical of the alerting response (Brod et al. 1959; Hilton 1982), whereas in the present study, young WE men and SA men showed no net pressor response and a trend for bradycardia. This disparity seems likely to reflect the unpleasantness of the stimulus: a lower frequency (1.5 kHz) in the present study vs 2 kHz (Edwards et al. 1998). Thus, low intensity sounds evoked vagal bradycardia, and higher intensity sounds evoked sympathetic tachycardia (Gianaros and Quigley 2001; Turpin et al. 1999). Variable HR responses were also reported to electric shocks, bradycardia being more common (Donadio et al. 2002).

### Limb vascular responses in individuals

The majority of young WE and SA men showed an increase in FVC (vasodilatation) at 5 s into each sound, but diverged thereafter. Thus, we divided them into “vasodilators” and “vasoconstrictors” according to the direction of change in FVC at 15 s when ABP and cutaneous vasoconstriction reach their peak (Edwards et al. 1998). This revealed the proportion of vasodilators was much smaller in SAs than WEs: ~ one-third vs two-thirds, respectively. However, importantly, within each ethnicity, the direction of change in FVC was highly consistent within individuals from the first to the fifth sound; just two vasodilator WEs showed habituation of the vasodilatation within session. As far as we are aware, this is the first evidence that forearm vascular responses to stress stimuli are reproducible on repetition in any ethnic group. However, it is reminiscent of the finding that when normotensive subjects were categorized on the basis of whether their MSNA decreased, or increased in response to an electric shock: 50–60% showed a decrease in MSNA, some showing habituation on repetition and the remainder showed an increase in MSNA. Furthermore, the direction of change in MSNA was reproducible between electric shock and mental stress and when electric shocks were repeated after several months (Donadio et al. 2002, 2012).

The question arises as to why more SAs than WEs were vasoconstrictors? The EDR (sweating) response and stress ratings for the sound stimuli were lower in SAs than WEs and SAs tended to report lower perceived stress scores for everyday life. Thus, the dominance of vasoconstrictors amongst SAs was not associated with greater responsiveness to, or perception of, mental stress. This was worth considering because some, but not all studies showed a positive correlation between intensity of perceived stress and increase in MSNA (Callister et al. 1992; Carter and Ray 2009; Donadio et al. 2012). In addition, the higher proportion of vasoconstrictors in SAs cannot be explained by family history of hypertension (Matsukawa et al. 1991) for a similar, small number SAs and WEs had hypertensive parents.

It seems more likely the disparity reflects the mechanisms that underlie the forearm vascular response. In many individuals, a sudden electric shock evoked a decrease in MSNA within one cardiac cycle (Donadio et al. 2002). Thus, a decrease in MSNA may well have contributed to the forearm vasodilatation that occurred at 5 s into the sound in the majority of WEs and SAs. Maintained forearm vasodilatation evoked by mental stress lasting 5 min has been reported to be accompanied by a long-lasting decrease in forearm MSNA (Halliwill et al. 1997), or by a progressive increase in forearm MSNA (Carter et al. 2005). Thus, it could be a higher proportion of SAs than WEs switched from a decrease in MSNA at 5 s, to an increase in MSNA by 15 s, so explaining the forearm vasoconstriction at 15 and 20 s. This seems unlikely given evidence that changes in MSNA evoked by short and longer-lasting stressors are consistent within individuals (Donadio et al. 2002, 2012).

An alternative explanation is that the disparity reflects a blunted contribution of endothelium-dependent dilatation to the forearm vascular response in SAs. The forearm vasodilatation of mental stress is highly dependent on shear stress-induced release of NO and the β_2_-adrenoceptor effect of adrenaline, which is NO-dependent (Dietz et al. 1994; Halliwill et al. 1997; Seddon et al. 2008). The NO-dependent component of stress-induced forearm vasodilatation was impaired in those with hypertensive parents, in hypertensives and in black African Americans (Cardillo et al. 1998a, b; Schlach et al. 2002; Khan et al. 2015). Thus, it is reasonable to propose that the majority of young SA men showed forearm vasoconstriction because their NO-dependent, and β_2_-adrenoceptor mediated dilatation was blunted. The few vasodilator SAs may have been those who showed greater stress-induced release of adrenaline and consequent NOS activation. This agrees with our finding that the vasodilators of both ethnicities showed larger sweating responses than the vasoconstrictors.

### Relationships between forearm vascular responses and ABP

In both WEs and SAs, sound-evoked increases in FVC in vasodilators were associated with repeated falls in ABP. This suggests that in these individuals, vasodilatation occurred in both forearms and possibly leg muscles and made a major contribution to the fall in ABP, for any change in HR was small and the alerting response includes generalized vasoconstriction except in muscle (Brod et al. 1959; Hilton 1982). On the other hand, the trend for ABP to increase repeatedly in the vasoconstrictor WEs and SAs suggests that they showed repeated vasoconstrictor responses in forearms and legs. Similarly, those in whom MSNA decreased in response to short and longer-lasting stressors showed a fall, or smaller rise in ABP than those in whom MSNA increased (Donadio et al. 2002, 2012). Indeed, the negative relationship between sound-evoked changes in ABP and FVC in young WE and SA men is consistent with the positive relationship between stressor-evoked changes in MSNA and ABP (Donadio et al. 2012).

From a wider perspective, the repeatability of FVC responses to stressor stimuli and the dominance of forearm vasoconstrictor responses in SAs indicate that more young SA, than WE men are exposed to the vasoconstrictor, pressor effects of environmental stressors in everyday life. Given the evidence that repeated stress-induced vasoconstriction promotes vascular hypertrophy and development of hypertension (Oparil et al. 2003) and that heightened pressor responsiveness to laboratory stressors is predictive of future CVD (Chida and Steptoe 2010), our findings indicate that young men of SA ethnicity are preferentially exposed to these effects. The presence of high levels of stress in everyday life would be expected to further exacerbate the tendency of SA men to develop hypertension, particularly as acute stress impairs endothelial vasodilator function (Ghiadoni et al. 2002).

### Study limitations

A larger study is required to indicate whether our findings hold for a wider population of young SA men, or encompass young SA women, while longitudinal studies would be required to test whether young vasoconstrictor SAs are indeed at higher risk of hypertension and CVD. Self-reporting of diet and physical activity allowed the study to be non-invasive and facilitated recruitment of subjects. However, blood samples would have allowed assay markers of oxidative stress, lipid profile and insulin resistance, while a standard exercise test would have allowed better comparison of functional capacity. Such tests may have indicated whether young SAs showed higher risk factors for CVD than WEs. Self-reporting may also have led to underestimation of family history of hypertension.

We focused on forearm vasodilator response to repeated sounds as this component of the alerting response is labile and a recognised marker for CVD; recordings of skin blood flow would have indicated whether the vasoconstrictor components also differ between SAs and WEs. Finally, as we used a short, physical stressor, it will be important to establish whether our findings can be extrapolated to longer lasting, psychosocial, or cognitive stressors.

## Conclusions

Our novel findings indicate that even in young adulthood, SA men resident in Europe who are physically active with no overt CVD, already show impaired reactive hyperaemia and forearm vasodilator responses to environmental stress relative to matched WE men. We suggest that this largely reflects blunted endothelium-dependent dilatation in young SA men and results in dominant forearm vasoconstrictor responses to stress. Blunted forearm vasodilatation may be an early indication of endothelium dysfunction that increases the risk of SA men developing hypertension and CVD in later life. Moreover, repeated limb vasoconstriction to stressors in everyday life may, over time, exacerbate the pressor response to stress in SA men, facilitating development of hypertension and further CVD.

## Abbreviations

ABP: Arterial blood pressure
ANOVA: Analysis of variance
CVD: Cardiovascular disease
EDR: Electrodermal response
FBF: Forearm blood flow
FMD: Flow-mediated dilatation
FVC: Forearm vascular conductance
HR: Heart rate
ICC: Intra-class correlation
MSNA: Muscle sympathetic nerve activity
NO: Nitric oxide
NOS: Nitric oxide synthase
PGs: Prostaglandins
PSS: Perceived Stress Scale
S: Sound
SA: South Asian
WE: White European
